# The Association between Video Game Time and Adolescent Mental Health: Evidence from Rural China

**DOI:** 10.3390/ijerph192214815

**Published:** 2022-11-10

**Authors:** Lili Li, Cody Abbey, Huan Wang, Annli Zhu, Terry Shao, Daisy Dai, Songqing Jin, Scott Rozelle

**Affiliations:** 1 School of Economics, Hangzhou Dianzi University, Hangzhou 310018, China; 2 Stanford Center on China’s Economy and Institutions, Freeman Spogli Institute of International Studies, Stanford University, Stanford, CA 94305, USA; 3 Department of Agricultural, Food and Resource Economics, Michigan State University, East Lansing, MI 48824-1039, USA

**Keywords:** adolescent health, video game time, mental health, rural China, developing countries

## Abstract

As digital devices like computers become more widely available in developing countries, there is a growing need to understand how the time that adolescents spend using these devices for recreational purposes such as playing video games is linked with their mental health outcomes. We measured the amount of time that adolescents in rural China spent playing video games and the association of video game time with their mental health. We collected data from primary and junior high schools in a poor, rural province in northwest China (*n* = 1603 students) and used the Depression, Anxiety, and Stress Scales (DASS-21) to measure mental health symptoms. The results indicated that the average video game time was about 0.69 h per week. There was a significant association between adolescent video game time and poorer mental health. Each additional hour of playing video games also increased the chance of having moderate or above symptoms. Moreover, boys and non-left-behind children had worse mental health if they played more video games. Our study contributes to literature on the links between recreational screen time and mental health, and it sheds light on an issue addressed by recent government legislation to limit the video game time of minors in China.

## 1. Introduction

The wide prevalence of adolescent mental health problems is a serious global public health concern. Mental illnesses affect 10 to 20% of the adolescent population worldwide [1]. Evidence shows a strong positive association between mental illness experienced during adolescence and negative life outcomes, such as lower educational attainment [2,3] and decreased work productivity [4]. Additionally, most mental health problems affecting adults originate in adolescence [5]. Because of this, it is important to identify risk factors of mental health symptoms early in life to better prevent (or reduce the seriousness of) mental illnesses from occurring later on [6].

The existing literature on the risk factors of adolescent mental health in low and middle-income countries (LMICs) is scarce, and the failure to address these issues could hinder social and economic development in these nations. Nearly 90% of the world’s children and adolescents reside in LMICs [7], yet mental health research in LMICs contributes to a small percentage of all published mental health research in the world [8]. The levels of mental health symptoms among adolescents may also be particularly severe in LMICs [9], where many young people are exposed to a wide range of risk factors, such as poverty and a lack of social support; there also is low access to mental health preventative and treatment services [10]. As the social-environmental factors that influence mental health can depend on societal context [11], there is a need to explore the determinants of adolescent mental health in different settings.

As greater shares of the adolescent population in LMICs gain access to the internet and electronic devices such as computers, one factor that may be playing an increasing role in the mental health of adolescents in these settings is exposure to digital media, such as video games, though past empirical evidence is almost exclusively limited to developed countries. Overall, studies that explore the association between video game time and adolescent mental health in high-income settings have produced mixed results. Some of these studies have identified a higher prevalence of adolescent mental health problems among those with higher amounts of video game usage [12], including more symptoms of anxiety and depression [13], as well as higher levels of stress [14]. Indeed, according to the American Psychiatric Association (APA), the unhealthy use of video games could lead to the loss of social relationships, lower levels of interest or engagement in other activities, and withdrawal symptoms such as irritability, anxiety, or sadness [15]. However, not all studies have found that video game use is associated with poorer mental health. One study with a sample of children aged 6–11 in western Europe did not identify any significant associations between self-reported mental health symptoms and video game usage [16], while another study conducted during the pandemic among adults in the U.K. found that time spent playing video games reduced self-reported levels of anxiety and stress, citing opportunities to socialize and cognitive stimulation as potential mechanisms [17]. Besides recreational video games, adolescents in LMICs are also becoming increasingly exposed to educational video games and gamified learning content, which studies have shown may increase student anxiety due to increased competition and interpersonal comparisons between students [18,19]. In contrast, a recent review found benefits of gamification on social-emotional skills and behavioral learning outcomes [20]. It is also possible that adolescents with certain characteristics may be more likely than others to be affected by the use of video games or other digital technologies; for instance, in one Canadian study, the association between longer screen time and poorer mental health was more significantly pronounced for girls than for boys [21].

One setting in particular where more research is needed on the link between video game use and adolescent mental health is rural China. Mental health problems are highly prevalent among adolescents in rural China, with approximately 20% at risk for depression and 68% at risk for any type of anxiety, including learning anxiety [22]. These figures are significantly higher than the 10–20% worldwide average [1]. At the same time, access to digital technologies and the internet is growing rapidly. In 2020, the internet penetration rate was 55.9% in rural China, compared to just 33.1% four years earlier [23]. Although there appear to be no recent data on the prevalence of video game use among rural children and adolescents specifically, official statistics indicate that over half of Chinese netizens nationwide played online games in 2020 [23]. The Chinese government recently passed legislation limiting online video game time among minors to no more than to one hour per day, three days per week [24]. However, there has been little research on how and to what extent video game time is related to adolescent well-being in China overall, and few if any studies have been conducted in rural areas [25].

Past studies in rural China generally have had at least one of several limitations. First, it appears that large-scale studies rarely explore how video game time is associated with symptoms of specific mental health disorders (e.g., depression, anxiety) using a standardized mental health measure, though one study involving 7729 rural adolescents in eastern China did identify a positive association between internet addiction (measured using the Internet Addiction Test) and depression (measured using the Zung self-rating depression scale) [26]. Second, studies did not identify whether certain subgroups of the population might be more prone to the negative effects of video game usage than others. For example, it has been estimated that there are over 61 million Chinese children living in rural communities (villages/townships) that are being cared for by a single parent, their grandparents, or other relatives due to large-scale parental migration to the cities [27]. These “left-behind children” (LBCs) lack the familial support that helps sustain good mental health and encourage healthy behaviors, and thus these adolescents may be particularly prone to the negative impacts of gaming [28]. A third drawback of past studies is that they conduct data collection via online surveys, which may result in a strong response bias when conducting studies that are related to video games [29].

In light of these drawbacks in the existing body of literature, the overall goal of this study is to measure the association between video game use time and mental health among adolescents in rural China. To meet this goal, the paper has the following specific objectives. First, we seek to describe the prevalence of video game use and mental health symptoms among sample adolescents. Next, we measure the association between the time that adolescents use video games and their mental health symptoms. Finally, we compare the associations between video game time usage and mental health across subgroups of adolescents with different sociodemographic characteristics, including the gender of the sample adolescents, left-behind status, and family assets.

## 2. Materials and Methods

### 2.1. Ethical Approval

Ethical approval for this study was granted by the Stanford University Institutional Review Board. Written consent forms were sent to parents or guardians of eligible adolescents prior to conducting the survey. Throughout the study, we adhered to the Declaration of Helsinki to maintain data privacy and confidentiality. As such, adolescents were prohibited from discussing their responses during or after the survey, and we deleted the names of the sample adolescents from all electronic files during data encryption.

### 2.2. Study Location and Sampling

The data presented in this study were collected in October 2020 from 30 rural schools located in northwest China’s Gansu province. The per capita yearly disposable income of rural households in Gansu province in 2019 was $1354 USD, which was significantly lower than the national average income of households in rural China ($2248 USD). Moreover, 52% of the population in Gansu were rural residents (that is, they lived in rural communities), compared to the nationwide average of 40% [30].

To select our sample, we obtained a list of all schools from the local education bureau and randomly selected 30 schools (20 primary schools and 10 junior high schools) for inclusion in our study. We conducted our study among fourth and fifth graders in each of the sample primary schools, as well as seventh and eighth graders in each of the sample junior high schools. There were two main reasons for sampling students from these grades. First, students in fourth grade or above had the necessary literary and numeracy skills to complete survey questionnaires; our pilot survey indicated that students in younger grades had trouble understanding and completing questions regarding demographic information. Second, sixth graders were excluded because they were busy preparing for middle school entrance exams, which made it difficult (or impossible) to gain school approval for inviting them to participate in surveys.

Due to financial constraints, we randomly selected two classes at most from the selected grades of each school. Specifically, if there were only one or two classes in a grade, all classes in this grade were selected. If there were more than two classes in a grade, we randomly selected two classes. Half of the students in each sample class who were present on the day of the survey were selected to participate in the survey. In total, we surveyed 1603 adolescents in 95 sample classes across the 30 schools. Adolescents that participated in the survey filled out a demographic survey, a mental health questionnaire, as well as questionnaires that measured covariates, including social support and bullying.

### 2.3. Measures

Adolescent mental health was measured by the 21-item self-report Depression Anxiety Stress Scales (DASS-21). The DASS-21 is a well-recognized, self-rating measure of depression symptoms, anxiety symptoms, and stress symptoms, which make up its three sub-scales. For each of the seven items on the three sub-scales, respondents indicate how well a statement applies to them during the previous week, using a 4-point Likert scale (0 = does not apply to me at all, 1 = applies to me to some degree, 2 = applies to me to a considerable degree, and 3 = applies to me very much, or most of the time). A total score for each of the three sub-scales is obtained by adding up the seven items and multiplying by 2 to calculate the final score. A higher score indicates a higher level of symptom severity. Cutoffs for moderate severity of each type of symptom are defined using established thresholds in the literature. Specifically, a score of 14 or higher indicates moderate levels of depression symptoms, 10 or higher indicates moderate levels of anxiety, and 19 or higher indicates moderate levels of stress symptoms. The DASS-21 has been used to measure mental health issues for respondents in different age groups and validated in many contexts around the world [31,32]. In China, the DASS-21 the scale has high validity and internal reliability (Cronbach’s alpha > 0.90) [31].

To measure the prevalence of video game use, adolescents were asked to report the number of minutes of video games they played on weekdays and weekends. Questions were separately asked for time spent playing games on the computer and time spent using a video game console. The total number of hours per week spent playing video games was then calculated by combining these times.

Adolescent social support was measured using the mainland Chinese version of the Multidimensional Scale of Perceived Social Support (MSPSS), which is a 12-item questionnaire which uses a Likert scale to assess perceived social support from three categories: family, friends, and significant others [33]. Each category comprises four statements pertaining to examples of support they might receive. For each statement respondents were asked to rate the extent to which it applies to them, with a possible range of 1 to 7: 1 (“Very Strongly Disagree”), 2 (“Strongly Disagree”), 3 (“Mildly Disagree”), 4 (“Neutral”), 5 (“Mildly Agree”), 6 (“Strongly Agree”), and 7 (“Very Strongly Agree”). The score for each category is derived by summing the four responses for its corresponding subscales and then dividing by 4 to obtain a mean value. The MSPSS has been used in many contexts internationally and its reliability and validity have been tested among adolescents in China (Cronbach’s alpha coefficient of family subscale = 0.86) [33,34]. According to the authors of the scale [35], “low social support” was defined as overall scores between 1 and 2.9.

Information on bullying victimization was collected using an international scale developed for the Progress in Reading and Literacy Study (PIRLS) known as the “Students Bullied at School” (SBS) scale. This scale has been used to describe the frequency of being bullied by peers among children across 52 countries and regions representing a variety of development and income levels; it has also been previously validated in China [36]. Following prior studies [37], responses were categorized into two groups based on whether or not respondents had experienced bullying on at least a monthly basis.

The survey also collected data on a number of other sociodemographic variables for each respondent. These include basic demographic characteristics, such as adolescent sex (male or female), age, and number of siblings. Information on whether adolescents were boarding at school (yes or no) was also collected. To measure the education level of each adolescent’s parents, we used high school attainment or above as a cutoff to create a dummy variable (>9 years education, yes or no). “Left-behind children” were classified as those adolescents whose parents both migrated out for work for more than six months in the past year. To measure socioeconomic status, the questionnaire asked whether or not their household owned seven selected items included in the National Household Income and Expenditure Survey to create a family asset index, which we categorized into quartiles [30].

### 2.4. Analysis

Our empirical analysis involved three main stages. First, we report the summary statistics of the sample, including means or proportions and standard deviations. We also conduct t-tests to compare video game usage among students with different characteristics. Second, we conduct ordinary least squares (OLS) regressions to measure the association between video game time and student mental health, adjusting for adolescent characteristics. Third, we run separate OLS regressions by adolescent subgroup, including gender (boy or girl), left-behind status (yes or no), and household income (top 25% or bottom 25%). All regressions controlled for class fixed effects, and standard errors are clustered at the school level.

Analyses were performed in Stata 16.1 [38]. Levels of statistical significance are reported at the 10%, 5%, and 1% levels.

## 3. Results

### 3.1. Summary Statistics

The summary statistics of the adolescents in our study are displayed in Table 1. Of the 1603 adolescents sampled, 55% were male and 45% were female, and the average age of adolescents was 12 years old. Fifty-nine percent (59%) of students attended primary school while 41% attended junior high, and 15% of students boarded at school. On average adolescents had one sibling, and 20% were left-behind children. About one quarter (24%) of the fathers of adolescents had received over nine years of education, compared to 15% of mothers. In terms of adolescent social environments, 11% of respondents had low perceived social support. Additionally, 43% were bullied at least monthly, and 70% did not often attend group activities at school. Finally, 30% of students had access to a computer at home.

### 3.2. Prevalence of Video Game Time and Mental Health Outcomes

**Figure 1:**
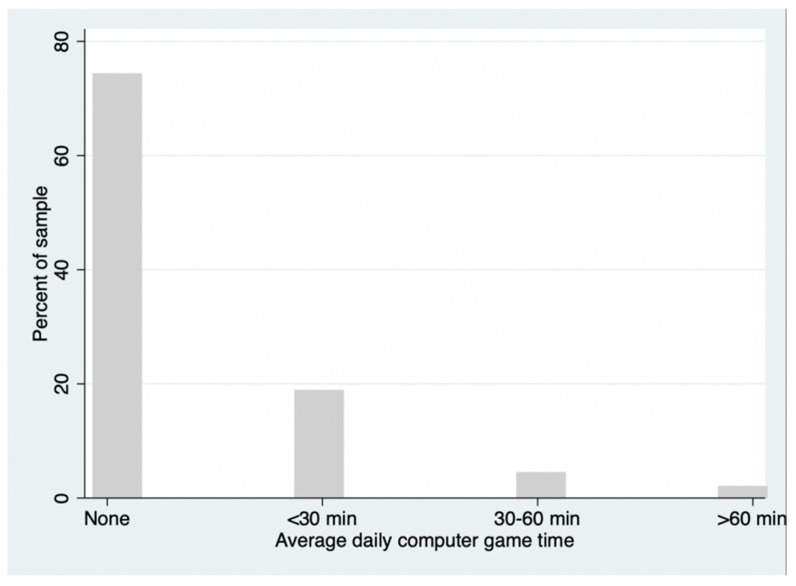
Histogram displaying average daily video game time of sample adolescents.

According to Table 1, adolescents played 0.69 h (or about 41 min) of video games per week, equivalent to about 6 min per day. Further breaking down the sample by daily video game time across different time thresholds (Figure 1, Appendix A Table A1), 75% of adolescents had no daily screen time at all, 19% of students had less than 30 min of screen time, 5% of adolescents had 30 to 60 min of screen time, and 2% of adolescents had daily video game time of over 60 min.

Table 2 explores differences in video game usage between different subgroups of sample adolescents. On average, boys, those aged 11 or older, and those enrolled in junior high school played 0.6 h (36 min) more per week than their peers (all significant at the 1% level, *p* < 0.001). Similarly, respondents that reported being bullied at least monthly played an average of 0.4 h (24 min) more than those bullied less often (*p* < 0.001). Similarly, adolescents who experienced low social support played 0.5 h (30 min) more than adolescents with higher social support (*p* < 0.001). However, several characteristics of adolescents did not correlate strongly with their video game time. This includes whether they board at school, their parents’ education levels, whether they were left-behind children or the only child, their family asset index, and whether they often attended group activities at school.

In terms of adolescent mental health, the mean DASS-21 sub-scale scores were 7.69 for depression symptoms, 9.53 for anxiety symptoms, and 10.88 for stress symptoms. Out of all our sample adolescents, 33% met the criteria moderate depression symptoms, compared to 51% for anxiety, and 29% for stress.

### 3.3. Association between Video Game Time and Mental Health Symptoms

Table 3 summarizes the overall correlation between video game time and mental health symptoms. Out of the entire sample, for every additional hour of video games played per week, the depression scores of the respondents increased by 0.41 points (*p* < 0.01), and anxiety scores increased by 0.33 points (*p* < 0.05). For all three of these outcomes, each additional hour of video games increased a respondent’s chance of having symptoms by one percentage point (for depression and stress, *p* < 0.05; for anxiety, *p* < 0.1). As this measure of video game time only accounted for game time using a computer and a video game console, in Appendix A Table A3 we added an additional control for whether the respondent used a mobile phone, and the results remained robust.

### 3.4. The Association between Video Game Time and Mental Health by Student Subgroups

The results of performing separate OLS regressions on video game time and mental health for different subgroups of student are displayed in Table 4. While the association between video game time and mean scores on the DASS-21 was not statistically significant for girls, it was significant for boys. Specifically, every additional hour of gaming increased the depression, anxiety, and stress subscale scores of male students by 0.44 points (*p* < 0.05), 0.38 points (*p* < 0.05), and 0.27 points (*p* < 0.1), respectively. The same was true when measuring the association between video game time and the share of adolescents with mental health symptom levels that were of moderate severity or above: while associations were not significant among girls, they were significant for boys. An additional hour of video game time increased the likelihood of having moderate depression symptoms by 2 percentage points (*p* < 0.01), and moderate stress symptoms by 1 percentage point (*p* < 0.05).

Similarly, when comparing left-behind children to their non-left-behind peers, there was a significant negative association between the video game time and mental health of non-left-behind children but not of left-behind children. An additional hour of gaming was linked with a 0.49-point (*p* < 0.05), 0.40-point (*p* < 0.05), and 0.32-point (*p* < 0.1) increase in the depression, anxiety, and stress subscale scores of non-left-behind children, respectively. The association with the likelihood of having moderate or above symptom levels of depression and stress was also significant (2 percentage points, *p* < 0.01; 2 percentage points, *p* < 0.05, respectively).

When comparing adolescents of higher and lower socio-economic status (family asset index), the associations between game time and mental health were more similar. For adolescents whose family asset index was in the top 50% of the sample, an additional hour of video game time per week was associated with a 0.43-point (*p* < 0.05) and 0.38-point (*p* < 0.1) increase in depression and anxiety scores, respectively; while for those whose family asset index was in the bottom 50%, their video game time was associated with a 0.47-point increase in depression. In terms of the likelihood of having moderate or above symptom levels of each condition, adolescents with an additional hour of video game time were 1 percentage point more likely to have depression scores and 2 percentage points more likely to have stress scores in the moderate or above range for those with higher family assets, while those with lower family assets were 2 percentage points more likely to have depression (*p* < 0.1) or anxiety scores above this threshold (*p* < 0.05).

## 4. Discussion

The research team explored the association between video game time and mental health among a large sample of adolescents (*n* = 1603) in a poor, northwestern province in rural China. The overall time adolescents spent playing video games was relatively low: the mean time per week was 0.69 h or 41 min per week (about 6 min per day), and 75% of adolescents did not report playing any video games. This is substantially lower than the prevalence of “frequent” gamers under the age of 18 according to recent national statistics in China, which estimated that 62.5% of children and adolescents frequently played video games nationwide in 2020 [23]. There are several possible reasons for why the prevalence of gaming was lower in our sample, among which include less access to digital devices in the sample region (only 30% of adolescents reported having access to a computer) as well as the narrower age range of the sample adolescents (the study participants in our sample were in grades 4, 5, 7, 8, compared to all school-aged students in the national report).

Although the overall duration of video game playing was relatively short, adolescents with certain characteristics played more frequently than others. Boys (0.94 h or about 57 min per day) played for over twice the duration of girls (0.38 h or about 23 min per day), reflecting gender differences in video game playing time in many other countries such as the U.S. [39] and Iran [40]; while junior high school students played for longer durations than primary school students (62 min versus 27 min, respectively). Two especially vulnerable subgroups of sample adolescents also had higher mean video game times than their peers, including those who were bullied monthly or more (about 62 min per week) and those with low levels of social support (about 67 min per week), highlighting the need to investigate how video game time is linked with adolescent mental health in rural China.

The primary finding of this study–that there was a significant association between video game time and worse adolescent mental health (according to the DASS-21)–contributes to an existing body of literature that has produced largely mixed results. For instance, one study of junior high school students in Iran found that both non-gamers (no video game time) and excessive gamers (defined as 10 h per week, or about 1.5 h per day) overall reported suffering poorer mental health based on General Health Questionnaire scores compared to low or moderate players [40]. Another systematic review found positive effects of casual (simple, easy-to-use) video games on a range of mental health outcomes, though this review only included studies with adult participants [41]. Meanwhile, a study in several European countries found that–after controlling for sociodemographic factors–there were no significant links between high video game usage (5 h per week) and child mental health as measured by the Strengths and Difficulties Questionnaire [16], while a meta-analysis of 101 empirical studies identified no significant link between video game use and adolescent mental health [42]. Other recent studies with panel data found no evidence that internet gaming disorder (IGD) contributed to more depression or anxiety symptoms among children and adolescents [43,44], though a third longitudinal study found that IGD predicted adolescent emotional distress [12].

There are several possible reasons for the lack of consistency between past results on the link between video game time and adolescent mental health. One potential reason is that the association between these two variables may depend on the gaming content or genre played [45], and the predominate genre played by sample respondents likely differs between studies and contexts. For instance, there is some evidence that video games whose primary purpose is not entertainment (“serious games”) may help reduce symptoms of anxiety and depression [41,46], while children and adolescents who play Massively Multiplayer Online Role-Playing Games (MMORPGs) may have a relatively high risk of addiction and mental health disorders. Additionally, while compulsive gaming behavior that interferes with one’s everyday activities (i.e., internet gaming disorder) is often linked with other mental health conditions [15,16], video games can also have benefits for mental health when used moderately, such as by increasing social interactions and reducing isolation [47], though one study among Canadian adolescents found no significant associations between video game time and mental health during the COVID-19 pandemic [48]. Future studies in rural China should consider how video game genres and different time thresholds (i.e., up to 1 h per day, 1–2 h per day, etc.) relate to adolescent mental health. According to a recent critical review on this topic [49], other factors to consider besides the amount of time played include motivation for use (why) and social context (who, what age, with whom, etc.) [49].

Our finding that video game time was more likely to have significant negative associations with the mental health of boys contrasts with those of two studies in the U.S. [39,50], which found significant associations between video game time and mental health for girls but not boys. One of these studies found that girls who had ever played video games before reported fewer depression symptoms and more aggressive tendencies compared to those who had not played any [39], and the authors suggested the possibility of reverse causality: video games may attract girls with certain characteristics (like higher externalizing symptoms and lower internalizing symptoms), while video games are popular among boys in general. The other study found that girls who played video games had higher levels of anxiety symptoms [50], positing that the competition and violence inherent in many video games is more stressful for girls than for boys, and that boys may be more likely to use games as a way to socially connect with others. These explanations are unable to explain the findings of our study in rural China, in which the association may have been stronger for boys rather than girls because girls overall played for a very short duration each week (about 20 min on average, compared to about one hour each week for boys).

Our subgroup analyses also revealed that videogame time was negatively associated with mental health outcomes for non-left behind children (but not so for left-behind children). While past literature relevant to this subgroup analysis between left-behind children and non-left behind children is very limited, studies generally point to better mental health outcomes overall among non-left behind children compared to their left-behind peers [51]. One potential explanation is that non-left-behind children–due to higher levels of emotional support at home–may be able to better identify mental health symptoms and therefore changes in their mental health status, as demonstrated by one study with a sample of rural school children from Sichuan, Anhui, and Henan, which reported that non-left behind children had higher levels of mental health literacy [52]. Non-left-behind children may also face a narrower range of risk factors in their social environment than left-behind children; therefore, they may be more sensitive to exposure to individual risk factors, like those associated with video game playing. Qualitative data collection approaches such as semi-structured interviews would aid in better understanding the reasons behind such differences between left-behind children and their peers.

Our study’s findings are timely considering the recent policy on video game playing in rural China, which restricted video game use among minors from 8 to 9 PM on Fridays, Saturdays, Sundays, and holidays [53]. The original motivation of the policy was to enhance the physical and mental wellbeing of minors and prevent video game addiction. In order to enforce the regulations, the Chinese government has required video game companies to use strategies like facial recognition to regulate log in [54], and more recently the government further banned the viewing of certain livestreamed video game content on platforms like Douyin and Kuaishou after a surge in popularity following the initial restriction on video game use [55]. It is yet unclear, how these measures have influenced how adolescents allocate their free time (e.g., shifting to more outdoor or physical activities, or other types of screen time), as well as whether or not these new restrictions have had any impacts on their mental health.

We acknowledge that there are several limitations to this study. First, because we used cross-sectional data in our analysis, we cannot establish causal links between video game time and mental health. Indeed, it is possible that a causal relationship can exist both ways; while video game usage may influence adolescent mental health, it is also possible that adolescents with worse mental health symptoms tend to play more video games [39]. As such, longitudinal studies should be conducted in the future to explore the causal relationship between these variables, if any. Second, our survey did not include mobile-based gaming as part of the calculation for video game time. Due to the limited access to devices such as computers and video game consoles in rural China and the higher availability of smartphones [23], it is possible that a significant share of the sample adolescents were playing video phones on their phones instead of (or in addition to) using computers and game consoles, which may explain the relatively low average video game time across our sample. Future studies in rural China should incorporate data about video game playing conducted using a wider range of devices. Third, in order to achieve a more nuanced understanding of the link between video game time and mental health, future studies should also explore heterogeneity across different types of game content as well as different game time thresholds.

## 5. Conclusions

This study examined the association between video game time and mental health among rural adolescents in China. We found that although the overall video game time prevalence was low (41 min per week), there was a significant link between video game time and worse mental health outcomes according to subscale scores on the DASS-21 as well as a higher prevalence of symptoms at the moderate or above level. In subgroup analyses, there were significant associations between video game time and mental health for boys and non-left-behind children but not so for girls and left-behind children. Our study contributes to the growing literature on the link between specific types of screen time and adolescent mental health. It also highlights the importance for future longitudinal studies to explore how video game time may affect adolescent mental health, which would be timely considering recent regulations on the video game use of minors in China.

## Figures and Tables

**Table 1:** Summary statistics of sample.

	Obs.	Mean	Std. Dev.
**Demographic and family characteristics**			
Female, 1 = Yes	1603	0.45	0.50
Age, years	1603	11.54	1.62
Board at school, 1 = Yes	1603	0.15	0.36
Junior high school, 1 = Yes	1603	0.41	0.49
Number of siblings	1603	1.15	0.84
Father has >9 years education, 1 = Yes	1603	0.24	0.43
Mother has >9 years education, 1 = Yes	1603	0.15	0.35
Left-behind child, 1 = Yes (both parents out migrant)	1603	0.20	0.40
Family asset index	1603	−0.01	1.24
Low social support, 1 = Yes	1603	0.11	0.31
Being bullied monthly or more, 1 = Yes	1603	0.43	0.49
Often attends group activities at school, 1 = Yes	1603	0.70	0.46
Have a computer at home	1603	0.30	0.46
**Measures of independent variables**			
Video game time, hours/week	1603	0.69	1.98
**Measures of outcome variables**			
Depression scores (DASS-21)	1603	7.69	8.55
Anxiety scores (DASS-21)	1603	9.53	8.37
Stress scores (DASS-21)	1603	10.88	8.60
Depression symptoms, 1 = Yes	1603	0.33	0.47
Anxiety symptoms, 1 = Yes	1603	0.51	0.50
Stress symptoms, 1 = Yes	1603	0.29	0.45

**Table 2:** Descriptive analysis of video game time: comparisons between different types of adolescents.

Social-Environmental and Demographic Factors			Video Game Time (Hours)		
		Obs.	Mean	Difference	*p*-Value
Gender	Female	715	0.382	−0.557 ***	0.000
	Male	888	0.939		
Age, years	≥11	852	0.972	0.600 ***	0.000
	<11	751	0.372		
Boards at school	Yes	240	0.839	0.175	0.207
	No	1363	0.664		
Junior high school	Yes	658	1.041	0.594 ***	0.000
	No	945	0.447		
Bullied monthly or more	Yes	683	0.896	0.357 ***	0.000
	No	920	0.538		
Father’s education level > 9 years	Yes	385	0.677	−0.018	0.875
	No	1218	0.695		
Mother’s education level > 9 years	Yes	233	0.788	0.114	0.418
	No	1370	0.674		
Left-behind child (both parents migrate)	Yes	316	0.645	−0.056	0.651
	No	1287	0.702		
Family asset index	Top 25%	397	0.865	0.234	0.111
	Bottom 25%	442	0.631		
Only child	Yes	219	0.463	−0.263	0.067
	No	1384	0.726		
Often attends group	Yes	1118	0.675	−0.052	0.626
activities at school	No	485	0.727		
Low social support	Yes	175	1.118	0.480 ***	0.002
	No	1428	0.638		

*** *p* < 0.01, ** *p* < 0.05, * *p* < 0.10.

**Table 3:** OLS regression of weekly video game time and adolescent mental health.

	Depression Scores	Anxiety Scores	Stress Scores	Depression Symptoms	Anxiety Symptoms	Stress Symptoms
Variables	(1)	(2)	(3)	(4)	(5)	(6)
Video game time, hours/week	0.41 ***	0.33 **	0.26	0.01 **	0.01 *	0.01 **
	(0.15)	(0.16)	(0.16)	(0.01)	(0.01)	(0.01)
Female, 1 = Yes	0.72 *	0.48	0.47	0.05 **	0.03	0.03
	(0.38)	(0.31)	(0.34)	(0.02)	(0.02)	(0.02)
Age, years	0.39	0.13	0.16	0.02	0.02	0.02
	(0.38)	(0.36)	(0.38)	(0.02)	(0.02)	(0.02)
Board at school, 1 = Yes	0.81	−0.58	−0.68	0.05	−0.06	−0.01
	(0.65)	(0.77)	(0.78)	(0.03)	(0.04)	(0.05)
Junior high school, 1 = Yes	−11.28 ***	−10.93 ***	−5.70 ***	−0.53 ***	−0.49 ***	−0.29 ***
	(1.14)	(0.96)	(0.94)	(0.06)	(0.05)	(0.05)
Father has >9 years education, 1 = Yes	0.11	−0.67	−0.07	−0.00	−0.05	−0.02
	(0.66)	(0.66)	(0.68)	(0.04)	(0.04)	(0.04)
Mother has >9 years education, 1 = Yes	−0.42	0.00	0.29	−0.01	0.02	0.03
	(0.72)	(0.78)	(0.78)	(0.04)	(0.05)	(0.03)
Left-behind child, 1 = Yes	0.25	0.50	0.18	−0.00	0.05	0.03
	(0.57)	(0.68)	(0.62)	(0.04)	(0.03)	(0.03)
Family asset index	−0.59 **	−0.40	−0.51 **	−0.02	−0.02	−0.02 *
	(0.22)	(0.24)	(0.22)	(0.02)	(0.01)	(0.01)
Number of siblings	0.48 **	0.55 ***	0.07	0.01	0.01	0.00
	(0.22)	(0.16)	(0.25)	(0.01)	(0.01)	(0.01)
Low social support, 1 = Yes	2.87 ***	−0.16	−0.36	0.12 **	−0.05	0.02
	(0.85)	(0.67)	(0.65)	(0.04)	(0.04)	(0.04)
Being bullied monthly or more, 1 = Yes	6.28 ***	6.12 ***	6.12 ***	0.33 ***	0.29 ***	0.26 ***
	(0.57)	(0.59)	(0.57)	(0.03)	(0.04)	(0.03)
Often attends group activities at school,	−1.28 **	−1.16 **	−0.97 *	−0.06 **	−0.08 **	−0.05 *
1 = Yes	(0.49)	(0.47)	(0.53)	(0.03)	(0.03)	(0.02)
Constant	6.73	12.10 ***	10.83 **	0.34	0.46 *	0.11
	(4.17)	(3.76)	(4.21)	(0.25)	(0.24)	(0.22)
						
Class fixed effect	Yes	Yes	Yes	Yes	Yes	Yes
Observations	1603	1603	1603	1603	1603	1603
R-squared	0.263	0.228	0.209	0.238	0.187	0.174

Robust standard errors in parentheses, clustered at school level. *** *p* < 0.01, ** *p* < 0.05, * *p* < 0.1.

**Table 4:** OLS regression of weekly video game time on adolescent mental health by groups.

	Depression Scores	Anxiety Scores	Stress Scores	Depression Symptoms	Anxiety Symptoms	Stress Symptoms
Variables	(1)	(2)	(3)	(4)	(5)	(6)
**Panel A, by student sex**						
**Female students**						
Video game time, hours/week	0.15	0.15	0.17	−0.00	0.02	0.02
	(0.24)	(0.21)	(0.35)	(0.02)	(0.01)	(0.02)
Controls	Yes	Yes	Yes	Yes	Yes	Yes
Class fixed effect	Yes	Yes	Yes	Yes	Yes	Yes
R-squared	0.381	0.303	0.304	0.334	0.269	0.273
**Male students**						
Video game time, hours/week	0.44 **	0.38 **	0.27 *	0.02 ***	0.01	0.01 **
	(0.17)	(0.18)	(0.16)	(0.00)	(0.01)	(0.01)
Controls	Yes	Yes	Yes	Yes	Yes	Yes
Class fixed effect	Yes	Yes	Yes	Yes	Yes	Yes
R-squared	0.271	0.254	0.218	0.254	0.197	0.188
**Panel B, by student left-behind status**						
**Left-behind students**						
Video game time, hours/week	0.03	0.03	0.02	−0.01	0.02	0.00
hours/week	(0.25)	(0.37)	(0.50)	(0.01)	(0.02)	(0.02)
Controls	Yes	Yes	Yes	Yes	Yes	Yes
Class fixed effect	Yes	Yes	Yes	Yes	Yes	Yes
R-squared	0.451	0.408	0.407	0.440	0.394	0.325
**Non-left-behind students**						
Video game time, hours/week	0.49 **	0.40 **	0.32 *	0.02 ***	0.01	0.02 **
	(0.19)	(0.17)	(0.18)	(0.01)	(0.01)	(0.01)
Controls	Yes	Yes	Yes	Yes	Yes	Yes
Class fixed effect	Yes	Yes	Yes	Yes	Yes	Yes
R-squared	0.276	0.247	0.222	0.262	0.201	0.202
**Panel C, by student family social economic status**						
**Family asset index top 50%**						
Video game time, hours/week	0.43 **	0.38 *	0.36	0.01 *	0.01	0.02 **
	(0.21)	(0.22)	(0.23)	(0.01)	(0.01)	(0.01)
Controls	Yes	Yes	Yes	Yes	Yes	Yes
Class fixed effect	Yes	Yes	Yes	Yes	Yes	Yes
R-squared	0.331	0.295	0.284	0.327	0.259	0.258
**Family asset index bottom 50%**						
Video game time, hours/week	0.47 *	0.41	0.28	0.02 *	0.02 **	0.01
	(0.23)	(0.27)	(0.20)	(0.01)	(0.01)	(0.01)
Controls	Yes	Yes	Yes	Yes	Yes	Yes
Class fixed effect	Yes	Yes	Yes	Yes	Yes	Yes
R-squared	0.296	0.281	0.255	0.276	0.236	0.211

Robust standard errors in parentheses, clustered at school level. *** *p* < 0.01, ** *p* < 0.05, * *p* < 0.1.

## Data Availability

We can upload and share data if needed.

## Appendix A

**Table A1:** Average daily video game time among sample adolescents (minutes).

	Obs.	Percentage of Sample
None	1194	74.49
<30 min.	302	18.84
30–60 min.	73	4.55
>60 min.	34	2.12

**Table A2:** OLS regression of daily video game time on adolescent mental health during school and weekend, respectively.

	Depression Scores	Anxiety Scores	Stress Scores	Depression Symptoms	Anxiety Symptoms	Stress Symptoms
Variables	(1)	(2)	(3)	(4)	(5)	(6)
						
**During weekdays:** Video game time	2.70 **	2.25 *	1.61	0.06	0.12 **	0.09 *
hours/day	(1.26)	(1.23)	(1.27)	(0.06)	(0.05)	(0.05)
Controls	Yes	Yes	Yes	Yes	Yes	Yes
Class fixed effect	Yes	Yes	Yes	Yes	Yes	Yes
R-squared	0.261	0.227	0.208	0.236	0.188	0.174
						
**During weekends:** Video game time	1.31 **	0.99 *	0.86 *	0.06 ***	0.02	0.04 **
hours/day	(0.48)	(0.51)	(0.50)	(0.01)	(0.03)	(0.02)
Controls	Yes	Yes	Yes	Yes	Yes	Yes
Class fixed effect	Yes	Yes	Yes	Yes	Yes	Yes
R-squared	0.260	0.226	0.208	0.239	0.185	0.173

Robust standard errors in parentheses, clustered at school level. *** *p* < 0.01, ** *p* < 0.05, * *p* < 0.1.

**Table A3:** OLS regression of weekly video game time on adolescent mental health, controlling for mobile phone use.

Variables	Depression Scores	Anxiety Scores	Stress Scores	Depression Symptoms	Anxiety Symptoms	Stress Symptoms
	(1)	(2)	(3)	(4)	(5)	(6)
						
Video game time, hours/day	0.47 ***	0.37 **	0.30 *	0.01 ***	0.01 *	0.02 **
	(0.15)	(0.15)	(0.16)	(0.01)	(0.01)	(0.01)
Controls	Yes	Yes	Yes	Yes	Yes	Yes
Class fixed effect	Yes	Yes	Yes	Yes	Yes	Yes
R-squared	0.265	0.231	0.211	0.239	0.191	0.175

Robust standard errors in parentheses, clustered at school level. *** *p* < 0.01, ** *p* < 0.05, * *p* < 0.1.
